# Supplementary material for: Censored Least Squares for Imputing Missing Values in PARAFAC Tensor Factorization
Source: bioRxiv. 2024 Jul 10:2024.07.05.602272. Preprint. [Version 1] doi: 10.1101/2024.07.05.602272 (PMC11257416; doi:10.1101/2024.07.05.602272)
Supplement: 1 [file NIHPP2024.07.05.602272V1-supplement-1.pdf]

bioRxiv preprint doi: https://doi.org/10.1101/2024.07.05.602272; this version posted July 10, 2024. The copyright holder for this preprint (which was not certified by peer review) is the author/funder, who has granted bioRxiv a license to display the preprint in perpetuity. It is made available under aCC-BY 4.0 International license.

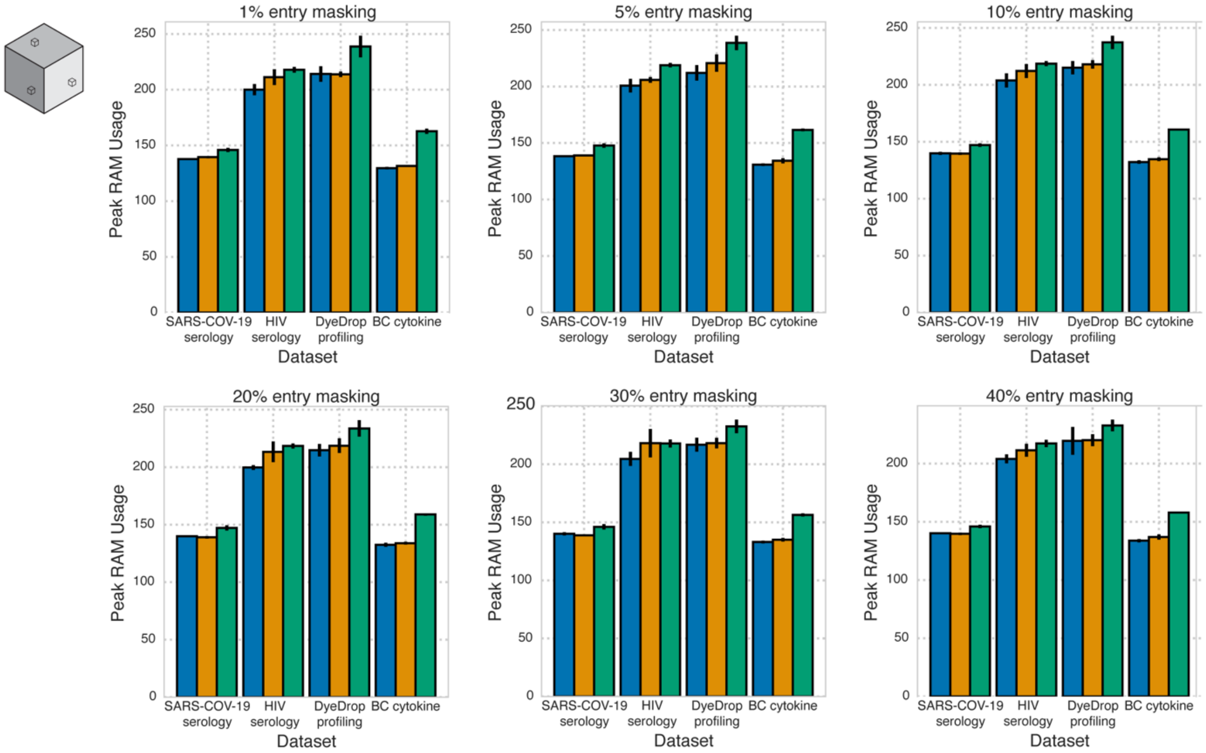

Fig. S1. Negligible differences exist in memory usage by algorithms across imputation types and extents. Median peak RAM Usage during **(a)** non-missing factorization and at **(b)** 5%, **(c)** 10%, **(d)** 20%, **(e)** 30%, **(f)** 40%, and **(g)** 50% random entrywise masking imputation.

bioRxiv preprint doi: https://doi.org/10.1101/2024.07.05.602272; this version posted July 10, 2024. The copyright holder for this preprint (which was not certified by peer review) is the author/funder, who has granted bioRxiv a license to display the preprint in perpetuity. It is made available under aCC-BY 4.0 International license.

Table S1. The direction of chordwise masking impacts imputation error but not selection of best solving algorithm. Median imputation errors at the best imputed rank are provided for chordwise masking along each chord for each solving algorithm at each imputation percentage.

Table S2. Optimal median imputation errors across entrywise imputation percentages. Median imputation errors at the optimal imputed rank under each solving algorithm at each entrywise masking percentage.

Table S3. Optimal median imputation errors across chordwise imputation percentages. Median imputation errors at the optimal imputed rank for under each solving algorithm at each chordwise masking percentage.

Table S4. Median time per iteration. Median seconds per iteration for each dataset for chordwise and entrywise masking combination (rows) for each solving algorithm at each drop percentage (columns). Values displayed are those at the best imputed rank.
